# GBA Mutations Influence the Release and Pathological Effects of Small Extracellular Vesicles from Fibroblasts of Patients with Parkinson’s Disease

**DOI:** 10.3390/ijms22042215

**Published:** 2021-02-23

**Authors:** Silvia Cerri, Cristina Ghezzi, Gerardo Ongari, Stefania Croce, Micol Avenali, Roberta Zangaglia, Donato A. Di Monte, Enza Maria Valente, Fabio Blandini

**Affiliations:** 1 Cellular and Molecular Neurobiology Unit, IRCCS Mondino Foundation, 27100 Pavia, Italy; cristina.ghezzi@mondino.it (C.G.); gerardo.ongari@mondino.it (G.O.); fabio.blandini@mondino.it (F.B.); 2 Department of General Surgery, Fondazione IRCCS Policlinico San Matteo, 27100 Pavia, Italy; stefania_croce186@yahoo.it; 3 Department of Clinical, Surgical, Diagnostic & Pediatric Sciences, University of Pavia, 27100 Pavia, Italy; 4 Department of Brain and Behavioural Sciences, University of Pavia, 27100 Pavia, Italy; micol.avenali@mondino.it; 5 Neurorehabilitation Unit, IRCCS Mondino Foundation, 27100 Pavia, Italy; 6 Parkinson’s Disease and Movement Disorders Unit, IRCCS Mondino Foundation, 27100 Pavia, Italy; roberta.zangaglia@mondino.it; 7 German Centre for Neurodegenerative Diseases (DZNE), 53175 Bonn, Germany; Donato.DiMonte@dzne.de; 8 Neurogenetics Research Center, IRCCS Mondino Foundation, 27100 Pavia, Italy; ezamaria.valente@unipv.it; 9 Department of Molecular Medicine, University of Pavia, 27100 Pavia, Italy

**Keywords:** Parkinson’s disease, glucocerebrosidase, extracellular vesicles, alpha-synuclein, GBA mutations, lipids, fibroblasts

## Abstract

Heterozygous mutations in the GBA gene, encoding the lysosomal enzyme glucocerebrosidase (GCase), are the strongest known genetic risk factor for Parkinson’s disease (PD). The molecular mechanisms underlying the increased PD risk and the variable phenotypes observed in carriers of different GBA mutations are not yet fully elucidated. Extracellular vesicles (EVs) have gained increasing importance in neurodegenerative diseases since they can vehiculate pathological molecules potentially promoting disease propagation. Accumulating evidence showed that perturbations of the endosomal–lysosomal pathway can affect EV release and composition. Here, we investigate the impact of GCase deficiency on EV release and their effect in recipient cells. EVs were purified by ultracentrifugation from the supernatant of fibroblast cell lines derived from PD patients with or without GBA mutations and quantified by nanoparticle tracking analysis. SH-SY5Y cells over-expressing alpha-synuclein (α-syn) were used to assess the ability of patient-derived small EVs to affect α-syn expression. We observed that defective GCase activity promotes the release of EVs, independently of mutation severity. Moreover, small EVs released from PD fibroblasts carrying severe mutations increased the intra-cellular levels of phosphorylated α-syn. In summary, our work shows that the dysregulation of small EV trafficking and alpha-synuclein mishandling may play a role in GBA-associated PD.

## 1. Introduction

Heterozygous mutations in the GBA gene, encoding the lysosomal enzyme glucocerebrosidase (GCase), are currently regarded as the strongest known risk factor for Parkinson’s disease (PD), after age [1]. Clinically, PD patients carrying GBA mutations (GBA-PD) tend to present an earlier age at onset, have faster disease progression, and have a greater occurrence of non-motor symptoms than patients not carrying mutations (non-mutated, NM-PD), but there is great phenotypic variability and some GBA-PD cases are indistinguishable from idiopathic PD [2,3,4,5]. An increasing number of studies have highlighted that such variability may relate, at least in part, to the severity of the underlying GBA mutations. Indeed, “severe” GBA mutations (e.g., L444P) are characterized by earlier PD onset, more severe progression, and a three-fold greater risk for dementia compared to “mild” mutations (e.g., N370S) [5,6,7]. In addition, patients carrying severe mutations show worse motor and non-motor manifestations, including more severe hyposmia and depression, as well as a more frequent occurrence of REM sleep behavior disorder [8].

Investigations carried out during the past several years have provided important clues on the relationship between GCase and PD. Nevertheless, the molecular mechanisms underlying the increased PD risk and the variable phenotypes observed in carriers of different GBA mutations are not yet fully elucidated. GCase converts glucosylceramide into ceramide and glucose, within the lysosome, and is therefore critically involved in sphingolipid metabolism. Mutations in the GBA gene may variably result in GCase protein degradation, disruptions in its targeting to lysosomes or reduction of enzyme activity, resulting in accumulation of glucosylceramide. An intriguing relationship has also been found between GCase activity and the protein alpha-synuclein (α-syn), a main constituent of the intraneuronal inclusions called Lewy bodies and Lewy neurites, which are typical of PD. A loss of GCase activity is associated with α-syn accumulation and the formation of pathological forms of the protein (e.g., aggregated and phosphorylated α-syn), possibly due to GCase-induced cellular lipid alterations and dysfunction of the autophagy–lysosomal system [9,10,11,12,13].

Extracellular vesicles (EVs) are a heterogeneous group of nanosized membrane vesicles that play an important role in intercellular communication. EVs have been proposed as potential contributors to the progression of neurodegenerative diseases, as they are responsible for the propagation of disease-associated proteins, lipids, and genetic material [14]. Interestingly, accumulating evidence links lysosomal function to EVs showing that perturbations of the endosomal–lysosomal pathway can affect EV release and composition [15,16].

In our study, we sought to investigate whether EVs may play a role in GBA-associated PD by exploring two hypotheses. First, we tested the possibility that GCase deficiency may promote the release of EVs. Secondly, we hypothesized that EVs derived from patients carrying GBA mutations may have detrimental effects on recipient cells and, in particular, trigger or enhance α-syn accumulation. Studies were performed using fibroblast cell lines from GBA-PD and NM-PD patients, as well as healthy control subjects (HC). We also investigated the impact of mutation severity on EV secretion and properties. Data showed that, independent of mutation severity, defective GCase activity enhances the EV release. Quite remarkably, EVs were capable of altering α-syn accumulation within recipient cells. This latter effect was mutation-dependent. Indeed, small EVs released from PD fibroblasts carrying severe GBA mutations increased the intra-cellular levels of phosphorylated α-syn suggesting a potential mechanism that could contribute to disease pathogenesis and more severe disease progression.

## 2. Results

### 2.1. GCase Deficiency Promotes the Release of Small Extracellular Vesicles

**Figure 1:**
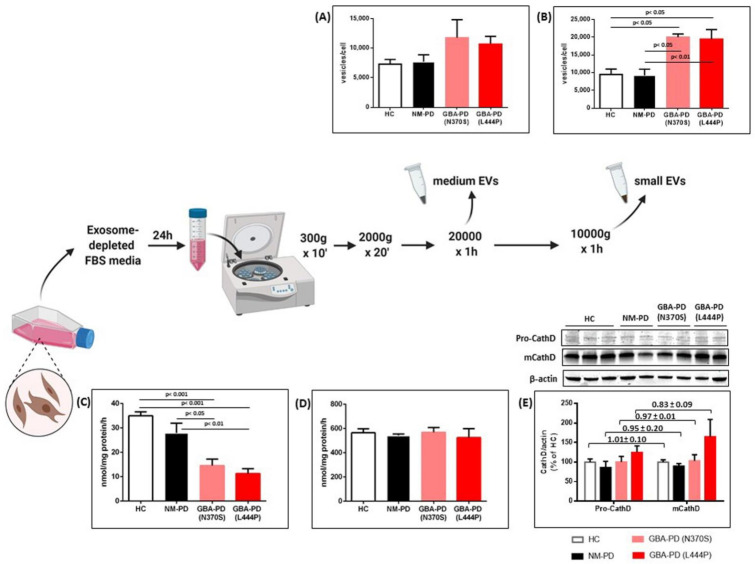
Assessment of EV release and lysosomal alterations in fibroblast cell cultures. (**A**,**B**) EVs were isolated by sequential centrifugation from surnatant of fibroblasts from non-mutated (NM-PD) and GBA-mutated (GBA-PD) PD patients, carrying mild (N370S) or severe (L444P) mutations, and healthy subjects (HC). Nanoparticle tracking analysis was used to determine the concentration of (**A**) medium and (**B**) small EV released. Data were expressed as number of vesicles secreted per cell. (**C**–**E**) Evaluation of (**C**) glucocerebrosidase and (**D**) cathepsin D (CathD) activity by fluorimetric assay, and (**E**) Western blot analysis of pro-CathD and mature CathD (mCathD) levels. The ratio between pro/mCathD was also reported in the bar graph. All data were expressed as mean ± SEM of three independent experiments (*n* = 2 for cathepsin D turnover).

In order to disclose a potential relationship between GBA-associated PD and EV release, secreted EVs were purified by ultracentrifugation from the supernatant of fibroblast cell lines derived from NM-PD and GBA-PD patients as well as HC, and quantified by nanoparticle tracking analysis (NTA). EVs are a heterogeneous population that includes medium EVs (mEVs; 200–1000 nm in size, sedimenting at <20,000 × g) and small EVs (sEVs, also known as exosomes, 50–200 nm in size, sedimenting at 100,000 × g) [17,18]. Since the two populations differ in terms of biogenesis, content, and potential functions, we investigated the impact of GBA mutations on the release of both mEVs and sEVs. Overall, NTA data indicate that GBA-PD-derived fibroblasts release a greater number of EVs as compared to fibroblasts from both NM-PD and HC (Figure 1A,B). This increase is especially evident for sEVs, whose number almost doubled in the GBA-PD group (Figure 1B, *p* < 0.05 (GBA-PD (N370S)) and *p* < 0.01 (GBA-PD (L444P)) vs. NM-PD; *p* < 0.05 vs. HC). No significant changes in size distribution of both EV subpopulations were found between groups (Table 1). Of note, the severity of GBA mutation did not affect either EV shedding nor size profile.

We then attempted to explore the potential mechanism underlying the increased EV release in GBA-PD fibroblasts. As expected, fibroblasts from GBA-PD patients showed approximately 50% residual GCase activity (Figure 1C), with a downward trend related to mutation severity. It has been recently demonstrated that cathepsin D (CathD)—a lysosomal aspartyl protease that contributes to normal GCase function by cleavage of prosaposin—would play a functional role in EV biogenesis and secretion [19]. Therefore, we assessed CathD expression levels (both the immature and mature form) and activity in our cell lines. Whereas no differences in the enzyme activity and maturation were observed in NM-PD and GBA-PD (N370S) fibroblasts as compared to control cells (Figure 1D,E), fibroblasts derived from patients carrying the GBA L444P mutation showed an upward trend in the CathD expression, especially for the mature form. While it is known that cathepsin defects may affect autophagy-mediated degradation, no evident alterations in common autophagic markers have been detected in our cell lines (Figure S1).

Since in our work GBA mutations have been shown to preferentially affect sEV release, we focused our attention on this vesicle subpopulation in the following experiments. The characterization of these vesicles is reported as Supplementary Materials (Figure S2).

### 2.2. Small EVs Derived from PD Fibroblasts Carrying Severe GBA Mutation Increase the Levels of Phospho-Ser129 α-Syn in Recipient Cells

**Figure 2:**
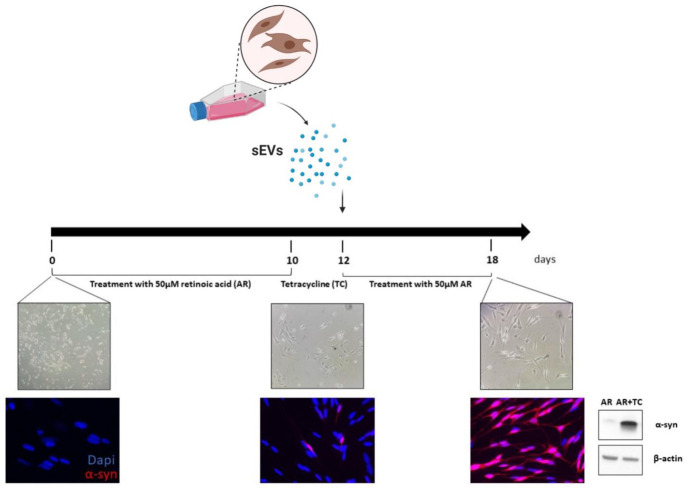
Inducible α-syn expression in differentiated SH-SY5Y cells: experimental design. Schematic representation of the timeline of SH-SY5Y differentiation and sEV treatment. Bright-field and fluorescent images (20× and 40× magnification, respectively) show the progressive development of a neuron-like phenotypes in these cell lines and the increase in α-syn expression after tetracycline treatment, as also confirmed by Western blot images.

To assess the ability of patient-derived sEVs to affect α-syn expression, we employed SH-SY5Y cells with inducible over-expression of human wild-type α-syn (hα-syn) [20]. Cells were first treated with retinoic acid to induce differentiation toward a neuronal phenotype, characterized by neuritic process extension. Then, cells were treated with tetracycline to trigger over-expression of hα-syn. Western blot and immunocytochemical analysis confirmed enhanced expression of soluble (monomeric, hereafter referred to as total) hα-syn in the lysates of tetracycline-treated cells, whereas the α-syn expression level in SH-SY5Y cells exposed to retinoic acid alone was barely detectable (Figure 2). In all subsequent experiments, potential changes in α-syn expression were then evaluated after the addition of tetracycline.

**Figure 3:**
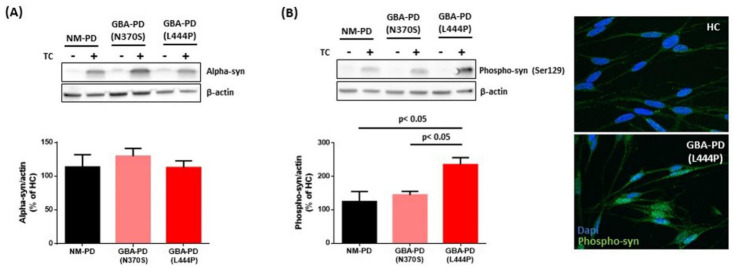
Effect of patient-derived sEVs on α-syn expression in SH-SY5Y cells. (**A**) Total α-syn levels in SH-SY5Y cells after treatment with sEVs derived from non-mutated (NM-PD) and GBA-mutated (GBA-PD) PD patients, carrying mild (N370S) or severe (L444P) mutations. (**B**) Expression levels and representative images of fluorescent signal of phospho-syn (Ser129) in SH-SY5Y cells after treatment with patient-derived sEVs. The expression of α-syn (total and phosphorylated form) is normalized with the housekeeping protein (β-actin) and reported as a percentage compared with α-syn expression in SH-SY5Y cells treated with healthy control (HC)-derived sEVs. All data were expressed as mean ± SEM of three independent experiments. Representative images of proteins blots are reported.

Hα-syn over-expressing SH-SY5Y cells were cultured for 5 days in the presence of sEVs isolated from the supernatant of fibroblasts of GBA-PD, NM-PD, and HC subjects. Levels of total and phosphorylated (phospho-Ser129) α-syn were assessed by Western blot. Exposure to sEVs, either from HC or PD patients (with or without GBA mutations), did not impact on cell survival (Figure S3) or total α-syn levels in SH-SY5Y cells (Figure 3A). To exclude that hα-syn over-expression induced in SH-SY5Y cells may mask further the increase elicited by EV treatment, we evaluated total α-syn after EV administration in SH-SY5Y cells exposed to retinoic acid alone (i.e., in the absence of tetracycline treatment). Again, no changes were observed and α-syn levels remained barely detectable in all groups (as shown in the blot pictures in Figure 3). Interestingly, treatment with GBA-PD-derived sEVs resulted in enhanced phospho-Ser129 α-syn in SH-SY5Y cells compared with cells receiving NM-PD vesicles. This effect was dependent on mutation severity, since a significant increase in phospho-Ser129 α-syn was primarily seen after administration of EVs isolated from PD fibroblasts carrying the L444P mutation compared both to NM-PD and to carriers of mild GBA mutation N370S (*p* < 0.05) (Figure 3B).

## 3. Discussion

GBA mutations, originally implicated in Gaucher’s disease, significantly increase the risk of developing PD. Together with the search for GBA-associated biomarkers, which could potentially predict the phenoconversion from GBA asymptomatic carriers to an overt PD phenotype, the identification of molecular mechanisms contributing to early onset as well as a more severe phenotype in carriers of GBA mutations still represents an unmet need.

We investigated the potential role of EVs in GBA-associated PD by focusing on EV release and their ability to induce pathological changes in recipient cells, in particular affecting α-syn levels. To address this point, we used fibroblast cell lines derived from patients carrying GBA mutations. We selected the two most common PD-associated mutations (severe L444P and mild N370S), which usually result in different clinical severity.

EVs have gained increasing importance in neurodegenerative diseases as “messengers of bad news”, since they transport and transfer pathological proteins (e.g., α-syn) thus promoting disease propagation [21]. Accumulating evidence suggests that, along with disease-associated proteins, EVs also carry a plethora of other proteins and lipids suggestive of pathological changes occurring in donor cells and potentially bearing a pathological role themselves [22,23,24,25].

In our study, we observed that the reduction in GCase activity detected in GBA-PD fibroblasts is associated with a considerable increase of EV release. Although it has been already reported that lysosome status influences EV secretion [15,16], to our knowledge, this is the first study addressing the effects of GBA-associated dysfunctions in PD patient-derived samples, by comparing the impact of mutation severity. A marked increase in released brain sEVs was observed after chronic inhibition of GCase activity by conduritol-B epoxide in A53T-syn transgenic mice [26]. Similarly, deletion of the GBA homolog dGBA1b in Drosophila caused a six-fold increase of EV concentration in the hemolymph (i.e., the Drosophila equivalent of blood) [27]. Of note, the increased release of EVs that we observed in GBA-PD fibroblasts specifically involved sEVs. Although medium and small EVs seem to share common biological functions, sEVs (also referred to as exosomes) have been demonstrated to play a relevant role in neurodegenerative diseases such as PD [28]. Exosomes are indeed the preferential mediators of the release and transmission of α-syn and toxic molecules (e.g., pro-inflammatory factors) in PD, probably representing one of the main actors in brain disease propagation [29,30,31]. Our data thus suggest that the selective increased release of sEVs induced by GCase deficiency could be a potential mechanism driving faster disease progression in GBA-associated PD.

To verify the hypothesis that GCase deficiency may also affect EV properties, in addition to their release, we assessed the ability of fibroblast-derived sEVs to alter α-syn levels in SH-SY5Y cells over-expressing this protein. While total α-syn remained unchanged in all experiments, exposure of SH-SY5Y cells to sEVs from GBA-PD patients increased the expression of phosphorylated α-syn. This effect was dependent on mutation severity, as the increase was driven by the GBA-PD (L444P)-derived sEVs, while no significant changes were triggered by GBA-PD (N370S)-derived sEVs. Since fibroblasts are basically an “α-syn-free system”, we can exclude that the effects observed in recipient cells may depend on the seeding properties of α-syn potentially carried by sEVs. We rather hypothesize that alterations in the lipid composition of GBA-PD-derived sEVs may account for the increased phospho-Ser129 α-syn levels observed in the recipient cells. Several studies demonstrated that alterations in lipid membrane metabolism, induced by GBA1 mutations, can promote conversion of α-syn into its insoluble form [32,33,34]. We recently demonstrated that the membrane of our GBA-PD (L444P) fibroblasts is characterized by an overall increase in sphingolipid levels and changes in the acyl chain length of ceramide, sphingomyelin, and hexosylceramide molecules, which are able to accelerate in vitro α-syn fibril formation [35]. Of note, the membrane of exosomes is enriched in these classes of lipids [36] and its composition can be affected by lysosomal dysfunction [15]. Grey et al. first examined α-syn aggregation in the presence of exosomes from cells without or with over-expressed α-syn, demonstrating that exosome lipids were sufficient for the protein aggregation and that the protein components of exosomes were not necessary for this effect to arise [37]. Recent studies showed that these vesicles directly transfer lipids from parent cells to recipient cells, triggering inflammatory and immune response or metabolism changes [38,39]. Therefore, GBA-PD(L444P)-derived exosomes may carry altered lipids to recipient cells, thus promoting pathological changes including α-syn mishandling. This phenomenon, together with the increased sEV release, may be part of the pathobiological mechanism that sustains the more severe disease progression observed in PD patients carrying this mutation. Clearly, testing this hypothesis will require an extensive lipidomic analysis of GBA-PD exosomes.

It should be also pointed out that, up to now, we did not observe the formation of α-syn aggregates in SH-SY5Y cells treated with GBA-PD sEV, probably due to the reduced duration of treatment. The increased expression of α-syn phosphorylated on Ser129, however, indicates the presence of a pathological modification that facilitates the process of fibril formation and insoluble aggregation [40,41]. The mechanism underlying this increase is still a matter of study and could involve CathD dysfunction, opening intriguing scenarios where impaired CathD expression could either be a secondary effect of exosomal lipid action, being a target protein for the ceramide [42], or could co-participate with lipids in promoting α-syn phosphorylation.

Lastly, as postulated by Thomas et al. [27] in the GCase-deficient Drosophila model, we cannot exclude that lipid alterations observed in GBA-PD (L444P) fibroblasts may also underlie the observed increased release of sEVs. Since ceramide is involved in sEV biogenesis [36], altered ceramide levels induced by GCase deficiency could affect sEV formation and trafficking. Whether the observed changes in CathD levels in fibroblasts carrying severe GBA mutations may contribute to these processes will require further investigation.

In summary, our work shows that mutations in the GBA gene promote dysregulation of sEV trafficking and conditions that favor α-syn fibrillation, such as phosphorylation on Ser129. These effects appear to be more marked in the presence of severe GBA mutation L444P. Further experiments on changes in EV lipid composition will be required to confirm current findings and to elucidate the EV-associated mechanisms by which mild and severe mutations differently drive disease progression and manifestations. Clarifying these mechanisms could promote the development of personalized therapeutic strategies able to prevent or delay disease progression by blocking excessive EV release or rescuing lipidic alterations.

## 4. Materials and Methods

### 4.1. Fibroblast Cell Culture

Fibroblasts were generated from skin biopsies of 9 PD patients carrying heterozygous GBA11 mutations (L444P *n* = 5; N370S *n* = 4), 6 sporadic PD patients without GBA mutations, and 5 healthy controls matched for age and gender. Fibroblasts were obtained from patients enrolled at the IRCCS Mondino Foundation and (two cell lines carrying the L444P mutation) from the Telethon Network of Genetic Biobanks. Before undergoing the skin biopsy, all subjects were genotyped in order to confirm their GBA mutation status using Sanger sequencing of the GBA gene [43]. Identified variants were confirmed by repeating amplification and sequencing with alternative primers. In all subjects, pathogenic variants and rearrangements in the other major PD-related genes (SNCA, LRRK2, PARK2, PINK1, and DJ-1) were previously excluded. The research protocol was approved by the Ethics Committee of the IRCCS Mondino Foundation and participants signed an informed consent. Fibroblasts were cultured in RPMI 1640 (Sigma-Aldrich, St. Louis, MO, USA) complemented with 1% streptomycin and penicillin (pen/strp) antibiotics and 10% fetal bovine serum (FBS, Sigma-Aldrich, St. Louis, MO, USA). Cells used in the experiments were grown and expanded in flasks up to a maximum of nine passages.

### 4.2. Enzyme Activity Assay

Protein lysates were obtained by resuspending fibroblast pellets in an ice-cold lysis buffer (CelLytic, Sigma-Aldrich, St. Louis, MO, USA) without phosphatase and protease inhibitors. After centrifugation, the supernatant was collected, and protein concentration was measured using a bicinchoninic acid protein (BCA) assay (Sigma-Aldrich, St. Louis, MO, USA).

GCase enzymatic activity in fibroblast cell lines was assessed in 5 μg of protein and determined by fluorometry using 4-methylumbelliferone as fluorochrome, as described before [44]. Fluorescence (excitation: 355 nm; emission: 460 nm) was measured using a microplate reader (CLARIOStar Plus, BMG LABTECH, Ortenberg, Germany) and results were reported as nmol of substrate/hour/mg protein.

Cathepsin D activity was assessed on fibroblast and SH-SY5Y lysates (1 μg of protein) with a fluorimetric assay kit (BioVision, Milpitas, CA, USA). Briefly, cell pellets were resuspended in 100 ul cathepsin D lysis buffer, incubated for 10 min on ice, and centrifuged at maximum speed for 5′. Fifty μL of samples were loaded together with negative controls in 96-well plates and incubated with a reaction mix (reaction buffer and substrate) at 37 °C for 1 h. Fluorescence (excitation: 328 nm; emission: 460 nm) was measured using a microplate reader (CLARIOStar Plus, BMG LABTECH, Ortenberg, Germany) and results were expressed as relative fluorescence unit.

### 4.3. EV Isolation and Quantification

EVs were isolated from conditioned media (CM) of fibroblast cell lines as described below. When cells were subconfluent (≈80%), the culture medium was removed, and cells were washed with PBS. New complete medium with EV-depleted bovine serum was added to the cells and incubated for 24 h. Following incubation, the CM was collected and centrifuged at 300 *g* × 10′, 2000 *g* × 20′ (Mikro 220R equipped with 1195-A rotor, Hettich, Tuttlingen, Germany) at 4 °C to eliminate dead cells and cellular debris/large apoptotic bodies, respectively. The CM was then centrifuged at 20,000 *g* × 60′, at 4 °C, to collect medium EVs (mEVs). A pellet containing mEVs was resuspended in 20 mM HEPES and underwent additional centrifugation. The supernatant was filtered through 13 mm sterile syringe filters with a 0.2 mm Supor (polyethersulfone ) membrane (Pall Corporation, Port Washington, NY, USA) and then centrifuged at 100,000 *g* for 60 min at 4 °C (Optima Max–XP equipped with TLA55 rotor, Beckman Coulter, Brea, CA, USA) to collect small EVs (sEVs). As for mEVs, the pellet containing sEVs underwent a further ultracentrifugation step in 20 mM HEPES. After this procedure, the pellet of vesicles was resuspended in 20 µL of 20 mM HEPES and used immediately for quantification experiments and SH-SY5Y treatment or processed for characterization experiments as detailed below.

Isolated EVs were analyzed by nanoparticle tracking analysis (NTA) to determine the particle number (for quantification and SH-SY5Y studies) and size distribution (for characterization experiments). A Nanosight NS300 instrument equipped with a 488 nm laser (Malvern Panalytical Ltd, Malvern, United Kingdom) was calibrated with polystyrene latex microbeads at 100, 200, and 400 nm prior to NTA. Resuspended vesicles were diluted with 20 mM HEPES to achieve between 20 and 100 objects per frame. Each sample was measured in quintuplicate at camera setting 15 with an acquisition time of 30 s and a detection threshold setting of 7. At least 200 completed tracks were analyzed per video. For each of the fibroblast cell lines, the analysis was repeated three times using different EV preparations. The NTA analytical software version 3.4 was used for capturing and analyzing the data. Data were expressed as the ratio between vesicle concentration/number of cells per flask at the time of isolation.

### 4.4. Transmission Electron Microscopy (TEM)

Exosome pellets were resuspended in sterile-filtered 100 μL PBS (Carlo Erba, Cornaredo, Italy) and a 5 μL drop of this suspension was absorbed on glow-discharge 300 mesh formvar/carbon copper grids; after 2 min, the grids were negatively stained with 2% uranyl acetate. Samples were imaged on an EFTEM Leo912 ab (Carl Zeiss, Jena, Germany) transmission electron microscope operating at 100 kV and digital images were recorded by a Proscan 1K slowscan CCD by iTEM Software (Olympus, Segrate, Italy).

### 4.5. SH-SY5Y Cell Culture and sEV Treatment

SH-SY5Y cells with tetracycline-inducible expression of human wild-type alpha-synuclein (hα-syn) were kindly provided by Dr. Philipp Kahle. For sEV treatment, SH-SY5Y cells were seeded at 500,000 cells/t75 flask and cultured in DMEM medium (Gibco) supplemented with 10% FBS, 1% (pen/strp), 5 mg/mL blasticidin, and 300 mg/mL zeocin (InvivoGen, San Diego, CA, USA). Cells were differentiated with 50 mM retinoic acid for 10 days. Then, α-syn expression was induced with an addition of 1 mg/mL tetracycline (InvivoGen, San Diego, CA, USA) for 3 days. After α-syn induction, fibroblast-derived sEVs (1.1 × 1010 particles/mL) were added to cell cultures and SH-SY5Y cells were treated for 5 days. The medium was changed on the third day and a fresh sEVs preparation was added to the medium. A pilot study was conducted to identify a physiologically relevant concentration of particles, in order to prevent excessive sEV burden potentially affecting cell viability. On day 5, cells were harvested, counted, and cell lysates analyzed by Western blot as described below. All experiments were repeated at least three times.

### 4.6. Western Blot Analysis

Protein lysates were obtained by resuspending fibroblast, SH-SY5Y cell or EV pellets in ice-cold lysis buffer (CelLytic, Sigma-Aldrich, St. Louis, MO, USA) containing protease and phosphatase inhibitors (protease inhibitor cocktail, 1:100; phosphatase inhibitor cocktail 1 and 2, 1:100, Sigma-Aldrich, St. Louis, MO, USA). The samples were kept on ice and placed on a shaker for 30 min, then centrifuged (16,000 *g* for 20 min at 4 °C). After centrifugation, the supernatant was collected, and protein concentration was measured by BCA assay (Sigma-Aldrich, St. Louis, MO, USA). Protein samples (20–30 μg) were resolved by electrophoretic run on a Mini-Protean^®^ TGX™ gel (AnyKD, Biorad, Hercules, CA, USA) and transferred to nitrocellulose membranes (Trans-Blot^®^ Turbo™ Transfer Pack, Biorad) through the Trans-Blot Turbo Transfer System (Biorad, Hercules, CA, USA). Membranes were blocked with a solution containing 5% BSA, 0.05% Tween-20 (Sigma) in PBS for 1 h and probed with the following primary antibodies: (a) anti-calnexin (1:2000, Proteintech, Manchester, United Kingdom), anti-flotillin (1:500, Proteintech, Manchester, United Kingdom), anti-TSG101 (1:500, Proteintech, Manchester, United Kingdom), and anti-GAPDH (1:2500, GeneTex, Irvine, CA, USA) for sEV characterization; and (b) anti-α-synuclein (1:2000, BD Biosciences, San Jose, CA, USA), anti-phospho-α-synuclein (S129) (1:500, Abcam, Cambridge, United Kingdom), and anti-β-actin (1:10,000, Santa Cruz, Dallas, TX, USA) for the evaluation of α-synuclein expression in SH-SY5Y cells. After washing, membranes were incubated with secondary antibodies (1:5000, Bio-Rad, Hercules, CA, USA) for 1 h at room temperature (RT) and visualized with enhanced chemiluminescence reagents (Biorad, Hercules, CA, USA) by using Azure600 (Azure Biosystems, Dublin, CA, USA).

Fluorescent near-infrared Odyssey^®^ scanner and software (Li-Cor Biosciences, Lincoln, NE, USA) were used for the detection of cathepsin D and autophagy-related protein. Membranes were blocked with the Odyssey blocking buffer and incubated overnight with the following primary antibodies: anti-cathepsin D (1:500, Santa Cruz, Dallas, TX, USA), anti-p62 (1:2000, Sigma-Aldrich, St. Louis, MO, USA), anti-beclin 1 (1:500, Cell Signaling Technology, Inc., Danvers, MA, USA), anti-LC3B (1:1000, Cell Signaling Technology, Inc., Danvers, MA, USA), and anti-β-actin (1:10,000, Santa Cruz, Dallas, TX, USA). As secondary antibodies, IRDye^®^ 700 and IRDye^®^ 800 (1:10,000) (Li-Cor Biosciences, Lincoln, NE, USA) were used.

The signal obtained from each protein was normalized with the corresponding signal obtained for the housekeeping protein. Data were expressed as a percentage compared to control samples in each membrane.

### 4.7. Statistical Analysis

Comparison between groups were carried out using one-way ANOVA followed by Dunn’s test. The minimum level of significance was set at *p* ≤ 0.05.

## Figures and Tables

**Table 1:** Average mean and modal size of small EV isolated from fibroblasts, obtained by nanoparticle tracking analysis. All data were expressed as mean ± SD.

	small EVs		medium EVs	
	Mode (nm)	Mean (nm)	Mode (nm)	Mean (nm)
HC fibroblasts	98.2±4.7	155.6±10.0	239.2±1.8	311.7±13.3
NM-PD fibroblasts	99.7±3.0	155.9±14.6	237.2±2.6	303.5±11.5
GBA-PD (N370S) fibroblasts	101.7±3.3	153.8±11.1	242.2±16.4	285.6±15.2
GBA-PD (L444P) fibroblasts	103.5±1.9	151.4±6.3	239.2±6.2	300.2±14.9

## Data Availability

The data presented in this study are openly available in Zenodo at 10.5281/zenodo.4556108.

## Supplementary Materials

The following are available online at https://www.mdpi.com/1422-0067/22/4/2215/s1.
