# Supplementary material for: GBA Mutations Influence the Release and Pathological Effects of Small Extracellular Vesicles from Fibroblasts of Patients with Parkinson’s Disease
Source: Int J Mol Sci. 2021 Feb 23;22(4):2215. doi: 10.3390/ijms22042215 (PMC7927041; doi:10.3390/ijms22042215)
Supplement: Supplementary file 1 [file ijms-22-02215-s001.zip › Supplementary files/Supplementary caption.docx]

**Figure S1. Characterization of fibroblast-derived sEVs. (A)** Representative particle-size distribution of sEVs. **(B)** Transmission electron microscopy of sEVs, scale bar = 100 nm **(C)** Western Blot for calnexin, flotillin and TSG101 in whole cell lysates (CL) and isolated sEVs.

**Figure S2. SH-SY5Y survival after treatment with sEVs.** All data were expressed as mean ± SEM. NT: non-treated, NM-PD: non-mutated patients, GBA-PD: PD patients carrying GBA mutations.

**Figure S3. Effect of patient-derived sEVs on Cathepsin D expression in SH-SY5Y cells.** Cathepsin D (CathD) levels in SH-SY5Y cells after treatment with sEVs derived from non-mutated (NM-PD) and GBA-mutated (GBA-PD) PD patients, carrying mild (N370S) or severe (L444P) mutations. The expression of CathD is normalized with the housekeeping protein (β-actin) and reported as percentage compared with CathD expression in SH-SY5Y cells treated with healthy control (HC)-derived sEVs. All data were expressed as mean ± SEM of three independent experiments. A representative image of proteins blot is reported.
