# Supplementary material for: GBA Mutations Influence the Release and Pathological Effects of Small Extracellular Vesicles from Fibroblasts of Patients with Parkinson’s Disease
Source: Int J Mol Sci. 2021 Feb 23;22(4):2215. doi: 10.3390/ijms22042215 (PMC7927041; doi:10.3390/ijms22042215)
Supplement: Supplementary file 1 [file ijms-22-02215-s001.zip › Supplementary files/Supplementary results.docx]

**Characterization of fibroblast-derived sEVs**

We confirmed the purity of our sEV preparation by using transmission electron microscopy and Western blot in addition to NTA. Particle size distribution analysis revealed that the sEVs ranged from 60 to 200nm in diameter, with a modal peak size comprised between 89 and 118nm (Figure S1A). Electron microscopy analysis confirmed the round shape morphology of the vesicles (Figure S1B). Moreover, western blotting demonstrated that EV markers TSG101 and Flotilin-1 were present in our vesicle preparation as expected. The purity of sEVs was confirmed by the lack of endoplasmic reticulum contaminant (calnexin) (Figure S1C). No evident difference between experimental groups were observed in the expression these markers.
