# Supplementary figures and images for: GBA Mutations Influence the Release and Pathological Effects of Small Extracellular Vesicles from Fibroblasts of Patients with Parkinson’s Disease
Source: Int J Mol Sci. 2021 Feb 23;22(4):2215. doi: 10.3390/ijms22042215 (PMC7927041; doi:10.3390/ijms22042215)

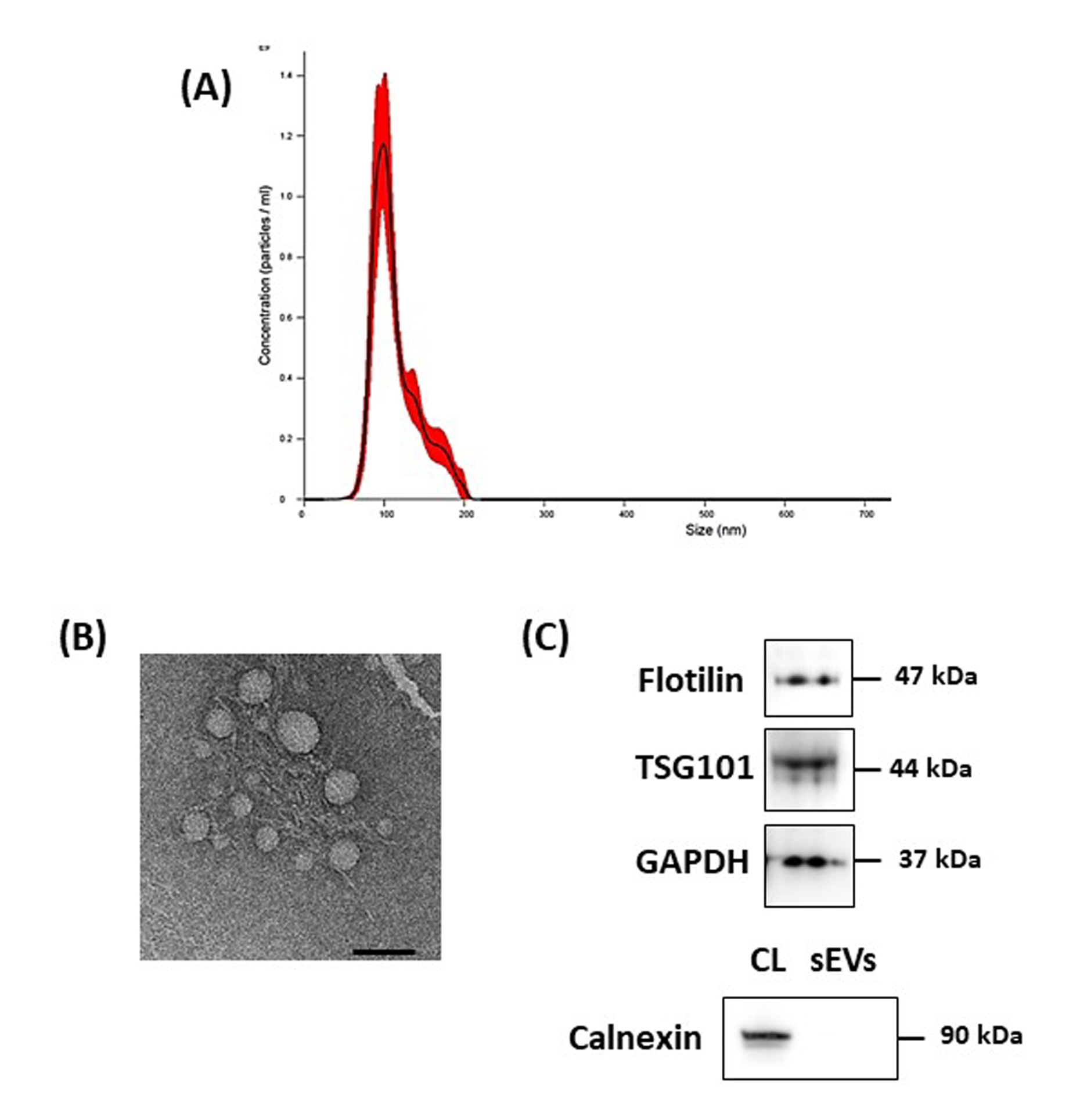

Supplement: Supplementary file 1 [file ijms-22-02215-s001.zip › Supplementary files/Figure S1rver2.jpg]

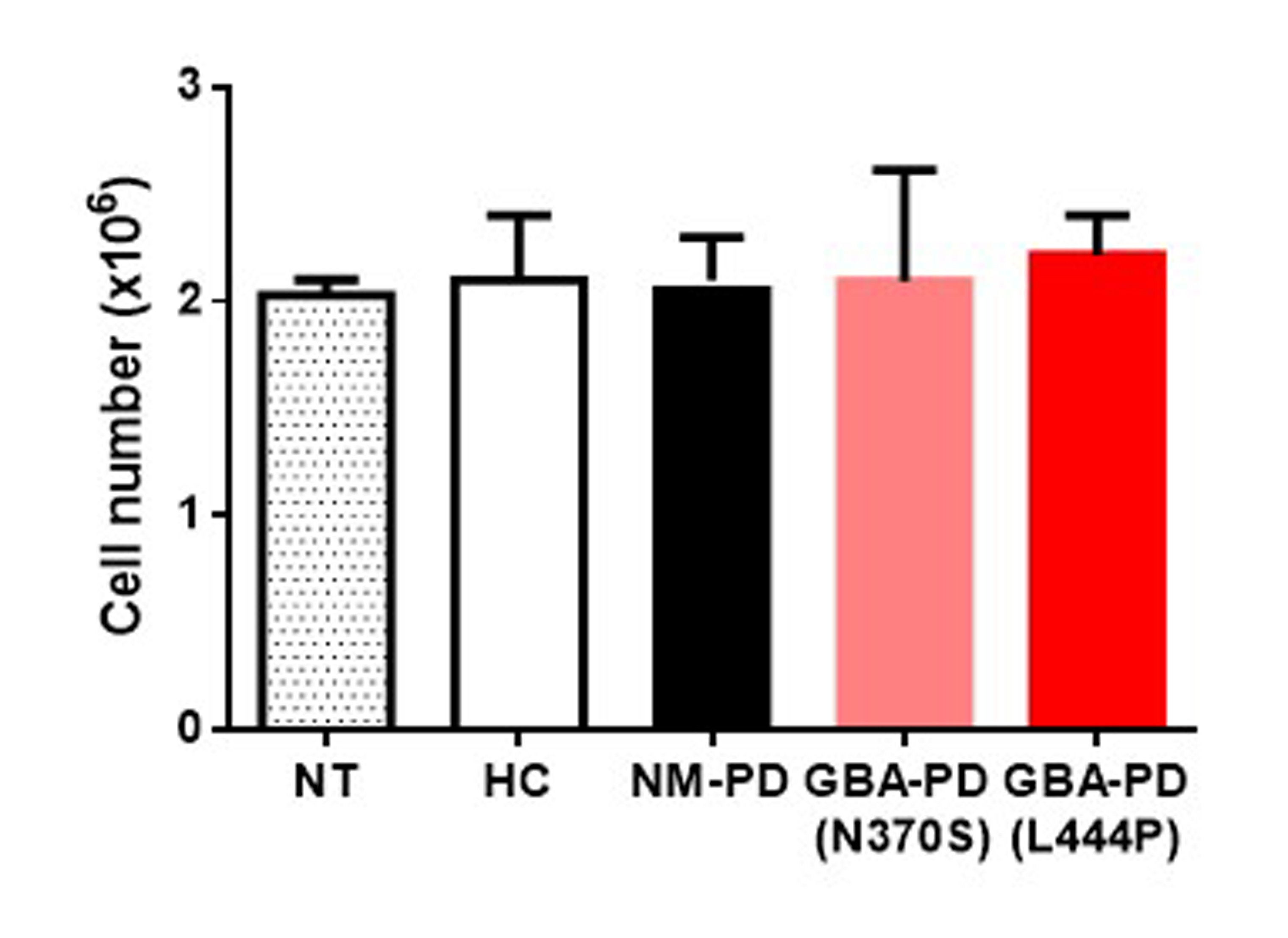

Supplement: Supplementary file 1 [file ijms-22-02215-s001.zip › Supplementary files/Figure S2r.jpg]

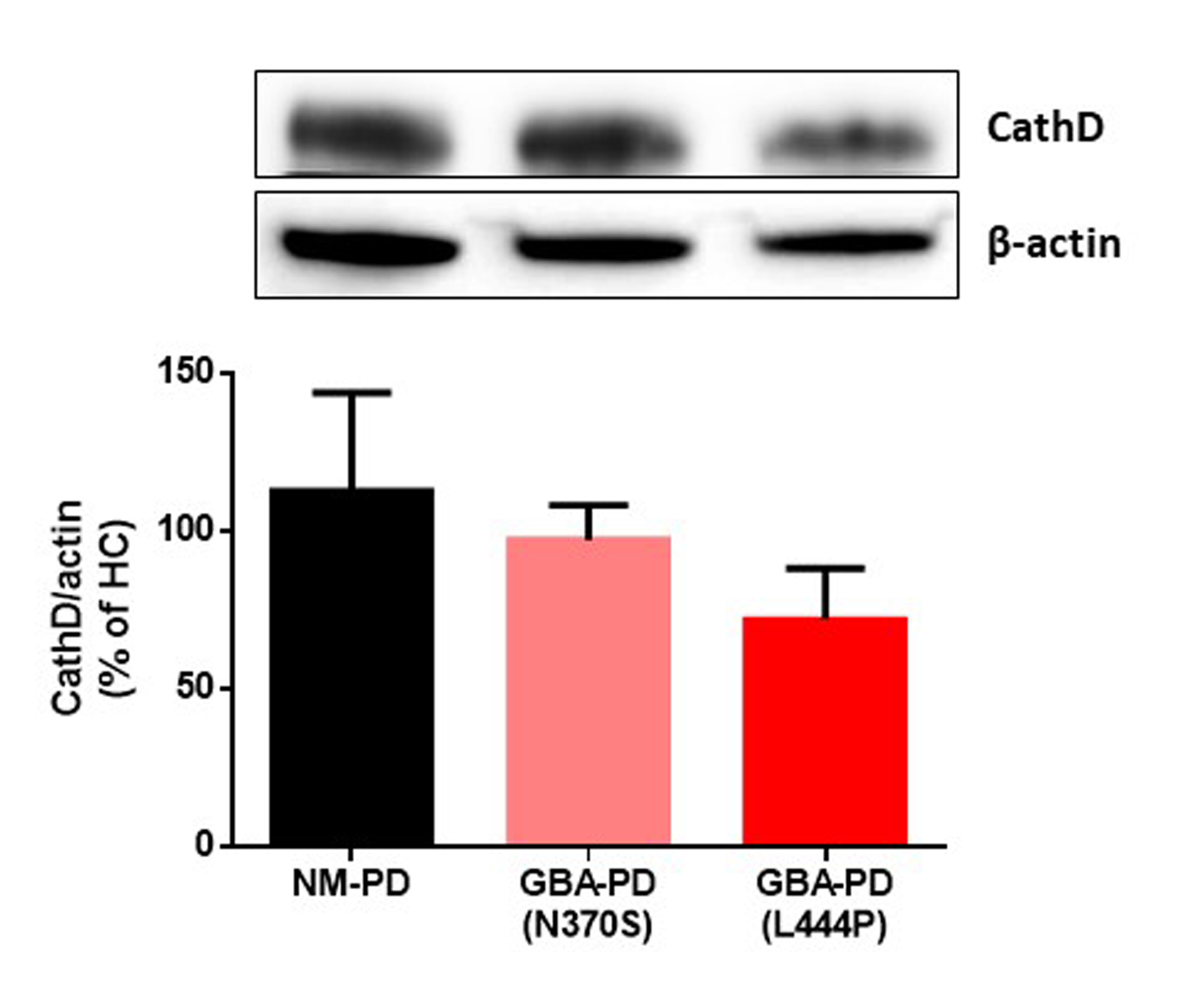

Supplement: Supplementary file 1 [file ijms-22-02215-s001.zip › Supplementary files/Figure S3.jpg]
